# Atherosclerotic Risk Factors and Risk of Myocardial Infarction and Venous Thromboembolism; Time-Fixed versus Time-Varying Analyses. The Tromsø Study

**DOI:** 10.1371/journal.pone.0163242

**Published:** 2016-09-16

**Authors:** Birgit Småbrekke, Ludvig Balteskard Rinde, Kristian Hindberg, Erin Mathiesen Hald, Anders Vik, Tom Wilsgaard, Maja-Lisa Løchen, Inger Njølstad, Ellisiv B. Mathiesen, John-Bjarne Hansen, Sigrid Brækkan

**Affiliations:** 1 K.G. Jebsen–Thrombosis Research and Expertise Center (TREC), Department of Clinical Medicine, UiT The Arctic University of Norway, Tromsø, Norway; 2 Division of Internal Medicine, University Hospital of North Norway, Tromsø, Norway; 3 Epidemiology of Chronic Diseases Research Group, Department of Community Medicine, UiT The Arctic University of Norway, Tromsø, Norway; 4 Brain and Circulation Research Group, Department of Clinical Medicine, UiT The Arctic University of Norway, Tromsø, Norway; 5 Department of Neurology, University Hospital of North Norway, Tromsø, Norway; Universitatsklinikum Hamburg-Eppendorf, GERMANY

## Abstract

**Background:**

Single measurements of modifiable risk factors may underestimate associations with outcomes in cohorts. We aimed to compare risk estimates of myocardial infarction (MI) and venous thromboembolism (VTE) by atherosclerotic risk factors during long follow-up using time-fixed analyses without and with correction for regression dilution and time-varying analyses.

**Methods:**

The study included 5970 subjects enrolled in the fourth survey of the Tromsø Study (1994/95). Blood pressure, lipid levels, body mass index (BMI), diabetes and smoking status were measured at baseline, and subjects still alive at the fifth (2001/02, n = 5179) and sixth (2007/08, n = 4391) survey were re-measured. Incident events of MI (n = 714) and VTE (n = 214) were recorded until December 2010. Time-fixed and time-varying Cox regression models were used to estimate hazard ratios (HR) for MI and VTE adjusted for age and sex.

**Results:**

Variations in BMI, blood pressure and lipid levels were small, and did not alter the risk estimates when time-varying analyses were compared to time-fixed analyses. For MI, variables that changed considerably over time yielded the greatest changes in risk estimates (HR for smoking changed from 1.80 (95% CI 1.55–2.10) to 2.08 (95% CI 1.78–2.42)). For VTE, only BMI was associated with increased risk in both time-fixed and time-varying analysis, but the risk estimates weakened in the time-varying analysis. Correction of time-fixed HRs with Rosner´s method tended to overestimate risk estimates compared to time-varying analysis.

**Comment:**

For MI and VTE, risk estimates based on baseline and repeated measures corresponded well, whereas correction for regression dilution tended to overestimate risks.

## Introduction

Arterial thrombotic disease (e.g. myocardial infarction [MI] and stroke) and venous thromboembolism (VTE) have traditionally been considered separate diseases with different pathophysiology, but during the last decade studies have supported a bidirectional association between them [1–4]. Whether the association between arterial and venous thrombosis is causal or mediated through shared risk factors remains uncertain. Of the traditional cardiovascular risk factors, only age, obesity and family history of MI have consistently been associated with VTE [5–10], whereas diabetes, hypertension and dyslipidemia have been associated with VTE in some [11–15] but not all [6, 16–18] studies. The majority of the studies that found an association between atherosclerotic risk factors and VTE were of a retrospective nature [11, 13–15], whereas most prospective studies reported no association [6, 16–18].

In conventional cohort studies, risk factor levels are usually assessed at the time of inclusion and related to outcomes occurring several years, or even decades, later. However, the status of a risk factor may change over time, and these changes usually become greater with time from exposure assessment. Both the effect of a risk factor (called time-dependent effect) and the value of the risk factor itself (called time-dependent covariate) can change over time. Random measurement errors, temporary fluctuations, and true changes in variables over time generally lead to regression dilution bias [19], a phenomenon that results in underestimation of the true association between exposure and outcome. As most atherosclerotic risk factors are modifiable, changes during follow-up may have influenced the risk estimates of MI and VTE in previous cohort studies. Thus, the absence of an association between atherosclerotic risk factors and VTE found in cohorts could potentially be explained by regression dilution.

Regression dilution bias is potentially a major limitation of prospective cohorts that could either be addressed by performing time-varying analysis or correct the risk estimates by a regression dilution ratio. When a variable is assessed within the same individual at different time points during the study period, time-varying analysis will allow for changes in exposure status during follow-up. If repeated measurements exists only for a subsample of individuals within a cohort, a regression dilution ratio can be calculated and used to correct the risk estimates from time-fixed analyses [20, 21]. Using this approach, a previous study reported that a single baseline measurement of cholesterol and diastolic blood pressure resulted in a respectively 47% and 76% underestimation of the association with coronary heart disease risk in the third decade of follow-up [22]. Another study reported that baseline assessment of disease risk underestimated the strength of the real associations by about one-third the first decade, about one-half the second decade, and about two-thirds the third decade [23]. However, it has been suggested that simple methods of correction for regression dilution bias may lead to overcorrection if the relationship between risk factor and disease is not short term [24].

In a prospective population-based cohort, we therefore aimed to investigate whether the use of repeated measurements of atherosclerotic risk factors influenced the risk estimates for VTE and MI compared to using baseline measurements only, with and without correction for the regression dilution bias. Secondly, we aimed to investigate whether the lack of association between atherosclerotic risk factors and VTE in previous long-term cohorts could be explained by regression dilution bias.

## Methods

### Study population

**Fig 1:**
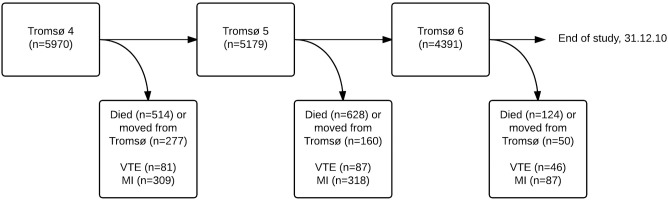
Study population. Study population recruited from The Tromsø Study, 1994–2010. Figure showing the common, “basic”, population in each analysis, and number of incident VTE and MI events (separate analyses for each outcome)

Participants were recruited from the fourth, fifth and sixth surveys of the Tromsø study (conducted in 1994–1995, 2001–2002 and 2007–2008, respectively). A detailed description of the Tromsø surveys has been published elsewhere [25]. In brief, the entire population (Tromsø 4) or parts of the population (Tromsø 5 and 6) aged ≥25 years living in the municipality of Tromsø, Norway, were invited to participate in these surveys. In Tromsø 4, all men aged 55–74 and women aged 50–74, as well as smaller (5–8%) random samples of other age groups were invited to a more extensive second examination. All subjects attending the second visit in Tromsø 4 were re-invited to attend Tromsø 5 and Tromsø 6 if they were still alive and living in the municipality of Tromsø. Subjects who attended the second phase of Tromsø 4 (n = 6861), as well as subjects attending the first phase of Tromsø 4 and the two subsequent visits (n = 418), were considered eligible for the present study. Subjects with VTE (n = 22) or MI (n = 306) prior to baseline, and subjects not officially registered as inhabitants of the municipality of Tromsø at baseline (n = 7), were excluded. Moreover, subjects who were re-invited but failed to attend one or more visits were excluded from follow-up (n = 974). Subjects who died (n = 1142) or moved (n = 437) between two subsequent visits were censored at the date of death or migration during follow-up. Thus, 5970 participants were included, of which 4391 attended all three surveys (Fig 1). The study was approved by the Regional Committee of Medical Health Research Ethics North Norway, and all participants provided informed written consent.

### Atherosclerotic risk factors

Information on atherosclerotic risk factors was collected by physical examinations, blood samples and self-administered questionnaires. Similar examinations, blood tests and questionnaires were repeated at each survey. Height and weight were measured with participants wearing light clothing and no shoes. Body mass index (BMI) was calculated as weight in kilograms divided by the square of height in meters (kg/m^2^). Blood pressure was measured three times with an automatic device (Dinamap Vital Signs Monitor) in a sitting position after two minutes of rest. The average of the two last readings was used in the analyses. Non-fasting blood samples were collected from an antecubital vein and total cholesterol, triglycerides and high-density lipoprotein (HDL) measured. Self-administered questionnaires were used to obtain information on diabetes, smoking (current smoker yes/no), physical activity (strenuous physical activity 1 or more hour per week) and education (over or equal to 15 years of education). Hypertension was defined as systolic blood pressure ≥140 mmHg, diastolic blood pressure ≥90 mmHg or current antihypertensive treatment. Overweight (BMI 25–29.9 kg/m^2^) and obesity (BMI ≥30 kg/m^2^) was classified according to the World Health Organization (WHO) definition [26], and hypercholesterolemia was defined as total cholesterol ≥6.5 mmol/L or self-reported use of lipid-lowering drugs. Low HDL cholesterol was defined as ≤1.03 mmol/L in men or ≤1.30 mmol/L in women, according to the National Cholesterol Education Program-Adult Treatment Panel III guidelines [27], as described elsewhere [28].

### Identification and validation of MI

All incident events of MI were identified by searching hospital and out-of hospital medical records, autopsy records and death certificates, and all possible events were validated by an independent end-point committee. The unique national 11-digit identification number allowed linkage to national and local diagnosis registries. Possible cases of MI were identified by linkage to the hospital discharge registry at the University Hospital of North Norway by searching for relevant International Classification of Diseases, as previously described [29] The hospital medical records were retrieved for case validation. MI events were validated according to modified World Health Organization MONICA/MORGAM criteria, including clinical signs and symptoms, findings in electrocardiograms, values of cardiac biomarkers and autopsy records when applicable [30]. Further, linkage to the National Causes of Death Registry at Statistics Norway allowed identification of fatal incident cases of MI that occurred as out-of hospital deaths, including deaths that occurred outside of Tromsø, as well as information on all-cause mortality. Information from death certificates was used to collect relevant information of the event from additional sources, including autopsy reports and records from nursing homes, ambulance services and general practitioners.

### Identification and validation of venous thromboembolism

As previously described [31], all incident VTE events were identified by searching the hospital discharge diagnosis registry, the autopsy registry and the radiology procedure registry at the University Hospital of North Norway. The University Hospital of North Norway is the only hospital in the region, and all hospital care and relevant diagnostic radiology is provided exclusively by this hospital. The medical record for each potential case of VTE was reviewed by trained personnel, and an episode of VTE was confirmed and registered as a validated VTE event when all of the following four conditions were satisfied: 1) confirmation by objective diagnostic procedures, including compression ultrasonography, venography, spiral computed tomography, perfusion-ventilation scan, pulmonary angiography or autopsy; 2) indication in the medical records that a physician diagnosed deep vein thrombosis or pulmonary embolism; 3) presence of clinical signs and symptoms consistent with deep vein thrombosis or pulmonary embolism; and 4) treatment with anticoagulants (heparin, warfarin), thrombolytic therapy or vascular surgery was required unless contraindications were specified [31]. VTE cases from the autopsy registry were recorded when the death certificate indicated VTE as the cause of death, or a significant condition associated with death.

### Statistical analysis

**Fig 2:**
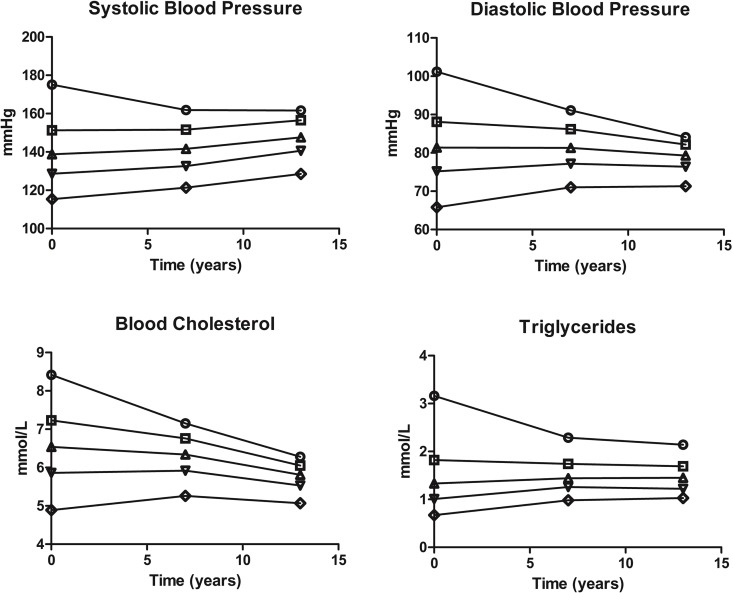
Intra-individual variability over time. Subjects were divided into quintiles at baseline in Tromsø 4 according to their baseline value of a certain risk factor. The mean value in each group is represented in the figure. Values were updated after approximately 7 and 13 years, in Tromsø 5 and Tromsø 6, respectively.

Statistical analyses were performed with STATA version 13.0 (Stata Corporation, College Station, TX, USA) and the figure showing intra-individual variability (Fig 2) was made using GraphPad Prism version 5.04 for Windows (GraphPad Software, San Diego California USA, www.graphpad.com). The significance level was set to 0.05. Follow-up time and risk estimates for VTE and MI were calculated separately. Atherosclerotic risk factors were measured at baseline (1994–1995), and subjects still living in the municipality of Tromsø at the fifth (2001–2002, n = 5179) and sixth (2007–2008, n = 4391) survey of the Tromsø study were re-measured. For each participant, person-years of follow-up were counted from the date of enrollment (1994–1995) to the date of an incident VTE or MI event (one analysis for each end-point), the date the participant died or moved from the municipality of Tromsø, or until the end of the study period (December 31, 2010), whichever came first. Age was used as time-scale and the entry and exit time was defined as the participants’ age at study enrollment and censoring event (MI, VTE, death, migration or end of study). We used three different approaches to calculate hazard ratios (HRs) of MI and VTE: (i) time-fixed analysis, (ii) correcting for time-dependent covariates using a regression dilution ratio, and (iii) time-varying analysis. In the first approach, we used a traditional Cox proportional hazard regression model that included baseline measurements from Tromsø 4. HRs with 95% confidence intervals (CI) for MI and VTE, adjusted for age as time scale and sex, were calculated. In the second approach, HRs calculated from traditional Cox proportional hazard regression models based on baseline measurements were corrected by a regression dilution ratio calculated by Rosner´s method for repeated measurements [32, 33]. This was done by multiplying the hazard coefficients of the normal time-fixed Cox regression model by the estimated regression dilution bias coefficient. The regression dilution bias coefficient was found as the inverse of the slope of the linear regression line of risk factor measurement of Tromsø 4 vs. Tromsø 5. The measurement time in Tromsø 5 was chosen (ignoring Tromsø 6) as the dependent value of the regression as it was approximately midway between the start and end of the study, as suggested by Clarke et al. [23]. In the last approach, a time-varying Cox proportional hazards regression analysis was used. Here, each participant contributed with one or more observation periods and each period lasted from one measurement until the next (i.e. from T4 to T5, from T5 to T6, and from T6 until the end of study the study period). The 5970 participants contributed with 15101 and 15403 observation periods in the MI and VTE analysis, respectively. The risk factors were updated at every measurement and used as time-dependent covariates in the analysis. The number of subjects included in the analyses of each risk factor varied slightly due to missing data for covariates (< 2% missing).

Joint modelling of repeated measurements and time-to-event data was considered, but found unsuitable due to convergence issues, as our dataset contained several participants with only one or two repeated measurements, particularly among those who experienced a VTE or MI event.

The proportional hazards assumption for the different risk factors was tested by evaluating the parallelism between curves of the log-log survivor function. The assumption was verified for all risk factors associated with VTE. For MI, the assumption was verified when stratifying by age (over and under 60 years old).

## Results

Among the 5970 participants, 741 subjects had an incident MI and 214 subjects an incident VTE during a median follow-up time of 15.7 years. The distribution of atherosclerotic risk factors in the three different surveys is shown in Table 1. The average value of BMI increased, whereas triglycerides and total cholesterol levels decreased from baseline and throughout the study. The mean age changed less than the time intervals between the studies as a consequence of people dying or moving from the municipality of Tromsø, and the proportion of men decreased over time due to a higher mortality rate among men. The most prominent changes were observed for the proportion of subjects with self-reported diabetes (increased from 2.4% to 7.1%), the proportion of smokers (decreased from 33.0% to 16.4%), and the proportion of physically active subjects which increased from 24.2% in 94–95 to 37.9% in 07–08. In addition, the proportion of subjects with hypertension increased from 49.6% in 94–95 to 67.5% in 07–08 (Table 1). Similar patterns were observed when the analyses were restricted to those who participated in all three surveys (S1 Table).

**Table 1:** Distribution of traditional atherosclerotic risk factors in the different surveys. In total 5970 were included in the study in 1994/95, and of these, 5179 and 4391 were re-measured in the 2001–02 and 2007–08, respectively.

The Tromsø Study	T4 (1994–1995)	T5 (2001–2002)	T6 (2007–2008)
Observations (n)	5970	5179	4391
Age, years	56.8 ± 11	62.9 ± 10	67.5 ± 10
Male sex	43.9	42.1	37.7
Systolic BP (mmHg)	141 ± 22	141 ± 21	145 ± 24
Diastolic BP (mmHg)	82 ± 13	81 ± 12	78 ± 11
Hypertension*	51.3 (3064)	56.8 (2941)	68.1 (3008)
Antihypertensive treatment	9.4 (560)	21.2 (1074)	35.2 (1511)
BMI (kg/m^2^)	25.7 ± 3.9	26.9 ± 4.1	27.0 ± 4.3
<25 kg/m^2^	46.5 (2775)	35.5 (1827)	34.3 (1503)
25–29.9 kg/m^2^	40.9 (2441)	44.7 (2302)	45.3 (1984)
≥30 kg/m^2^	12.5 (747)	19.9 (1023)	20.4 (894)
Triglycerides (mmol/L)	1.59 ± 1.01	1.53 ± 0.87	1.49 ± 0.81
Total cholesterol (mmol/L)	6.58 ± 1.27	6.28 ± 1.16	5.73 ± 1.13
Hypercholesterolemia †	51.9 (3097)	50.5 (2614)	46.9 (2058)
Lipid lowering drugs	1.8 (83)	12.3 (617)	23.3 (992)
HDL cholesterol (mmol/L)	1.56 ± 0.43	1.49 ± 0.40	1.57 ± 0.46
≥1.03 (♂) or ≥1.30 (♀) mmol/L	84.0 (5000)	78.6 (4051)	83.4 (3616)
<1.03 (♂) or <1.30 (♀) mmol/L	16.0 (954)	21.4 (1104)	16.6 (722)
Self-reported diabetes	2.4 (144)	4.2 (213)	7.1 (302)
Smoking	33.0 (1967)	25.6 (1328)	16.4 (719)
Physical activity |	24.2 (1492)	33.5 (1367)	37.9 (1383)
Education ⁞	20.0 (1191)	20.6 (1064)	24.1 (1037)

Values are % (n) or mean±SD. BP indicates blood pressure; BMI, body mass index; HDL, high-density lipoprotein.

*Hypertension: systolic BP ≥140 or diastolic BP ≥90 or use of antihypertensive medicine

**†**Hypercholesterolemia: total cholesterol ≥ 6.5 or use of lipid-lowering drugs

|Hard physical activity 1 hour or more every week

⁞Over/equal to 15 years of education (corresponding to 3 years in university or academy)

Even though the overall changes in blood pressure and blood lipids were small, we observed large differences when subjects were divided into quintiles according to their baseline value (Fig 2). In general, the groups in the lowest and highest quintiles at baseline changed the most, and shifted towards the overall mean. This was a result of both regression towards the mean, where the most extreme values tend to normalize over time, and measurement errors.

Table 2 shows hazard ratios for MI by the different atherosclerotic risk factors. All risk factors except one subgroup of BMI (BMI 25–29.9 kg/m^2^) were significantly associated with MI. For continuous variables, the differences were generally very small when the risk estimates based on baseline measurements (i.e. time-fixed analysis) were compared with those from repeated measurements (i.e. time-varying analysis). The largest differences between the two methods were observed for the categorical variables, and were particularly prominent for the variables that changed most at the population level, i.e. diabetes, smoking and physical activity. For MI, the risk estimates associated with diabetes changed from 2.94 (95% CI 2.20–3.92) to 2.11 (95% CI 1.63–2.72), smoking from 1.80 (95% CI 1.55–2.10) to 2.08 (95% CI 1.78–2.42) and physical activity from 0.74 (95% CI 0.61–0.89) to 0.61 (95% CI 0.50–0.74). Other risk estimates that changed considerably when comparing the two methods were those associated with hypertension, where the HR changed from 1.90 (95% CI 1.61–2.25) to 1.72 (95% CI 1.44–2.06) and obesity (BMI ≥30 kg/m^2^), where the HR changed from 1.43 (95% CI 1.15–1.79) to 1.28 (95% CI 1.04–1.57). The regression dilution correction by Rosner´s method consistently overestimated the risk estimates compared with the time-varying analyses (Table 2).

**Table 2:** Age (as time scale)- and sex-adjusted hazard ratios (HR) with 95% confidence intervals (CI) for the risk of myocardial infarction (MI) by traditional atherosclerotic risk factors using three different approaches; time-fixed model, time-varying model and correction for regression dilution through Rosners´s method. The Tromsø Study 1994–2010.

Risk factors	Time-fixed Cox-model	Time-varying Cox-model	Time-fixed model corrected by Rosner´s method
	HR (95% CI)	HR (95% CI)	HR (95% CI)
Male sex	2.46 (2.11–2.87)	2.46 (2.11–2.87)	2.46 (2.11–2.87)
Systolic BP (per 15 mmHg increase)	1.31 (1.23–1.37)	1.23 (1.18–1.29)	1.54 (1.43–1.67)
Diastolic BP (per 10 mmHg increase)	1.26 (1.18–1.32)	1.24 (1.18–1.32)	1.49 (1.35–1.65)
Hypertension*	1.97 (1.66–2.35)	1.73 (1.44–2.07)	3.69 (2.65–5.14)
BMI, 3 units increase (kg/m^2^)	1.14 (1.08–1.21)	1.10 (1.04–1.16)	1.15 (1.08–1.21)
<25 kg/m^2^	Ref.	Ref.	Ref.
25–29.9 kg/m^2^	1.15 (0.98–1.35)	1.05 (0.89–1.24)	1.17 (0.96–1.43)
≥30 kg/m^2^	1.43 (1.15–1.79)	1.28 (1.04–1.57)	1.25 (1.09–1.44)
Triglycerides (mmol/L)	1.21 (1.15–1.28)	1.23 (1.16–1.31)	1.49 (1.33–1.67)
Total cholesterol (mmol/L)	1.22 (1.14–1.29)	1.23 (1.16–1.31)	1.43 (1.28–1.60)
Hypercholesterolemia †	1.44 (1.24–1.68)	1.42 (1.22–1.65)	1.90 (1.45–2.50)
HDL cholesterol (mmol/L) ‡	0.78 (0.70–0.86)	0.78 (0.70–0.86)	0.71 (0.62–0.81)
≥1.03 (♂) or ≥1.30 (♀) mmol/L	Ref.	Ref.	Ref.
<1.03 (♂) or <1.30 (♀) mmol/L	1.48 (1.24–1.78)	1.34 (1.12–1.61)	2.06 (1.47–2.89)
Self-reported diabetes	2.94 (2.20–3.92)	2.11 (1.63–2.72)	3.17 (2.33–4.32)
Smoking	1.80 (1.55–2.10)	2.08 (1.78–2.42)	2.28 (1.85–2.82)
Physical activity |	0.74 (0.61–0.89)	0.61 (0.50–0.74)	0.40 (0.22–0.70)
Education ⁞	0.54 (0.43–0.69)	0.55 (0.43–0.69)	0.54 (0.43–0.69)

*Hypertension: systolic BP ≥140 or diastolic BP ≥90 or use of antihypertensive medicine

**†**Hypercholesterolemia: total cholesterol ≥ 6.5 or use of lipid-lowering drugs

**‡**HDL: per 0.5 mmol/L decrease

|Strenuous physical activity 1 hour or more every week

⁞Over/equal to 15 years of education (corresponding to 3 years in university or academy)

Hazard ratios of VTE according to the different atherosclerotic risk factors are shown in Table 3. In general, there were only small differences between the risk estimates based on the time-fixed analysis and those calculated with the time-varying analysis. In the time-fixed model and in the model corrected by Rosners´s method, BMI and hypertension was significantly associated with VTE. However, the association between hypertension and VTE disappeared when adjusting for BMI in addition to age and sex with HR of 1.24 (95% CI 0.92–1.69) and 1.52, (95% CI 0.85–2.73) in the time-fixed model and in the model corrected by Rosner´s method, respectively. When using time-varying analysis, only BMI was significantly associated with VTE (HR 1.21, 95% CI 1.10–1.33, per 3 kg/m^2^ increase for BMI). For BMI as a continuous variable and for the overweight subgroup (BMI 25–29.9 kg/m^2^), the Rosner correction gave slightly higher risk estimates than the time-varying analysis, whereas for obesity (BMI ≥30 kg/m^2^) the Rosner correction gave a lower risk estimate than the time-varying analysis.

**Table 3:** Age (as time scale)- and sex-adjusted hazard ratios (HR) with 95% confidence intervals (CI) for the risk of venous thromboembolism (VTE) by traditional atherosclerotic risk factors using three different approaches; time-fixed model, time-varying model and correction for regression dilution through Rosners´s method. The Tromsø Study 1994–2010.

Risk factors	Time-fixed Cox-model	Time-varying Cox-model	Time-fixed model corrected by Rosner´s method
	HR (95% CI)	HR (95% CI)	HR (95% CI)
Male sex	1.22 (0.94–1.60)	1.22 (0.94–1.60)	1.22 (0.94–1.63)
Systolic BP (per 15 mmHg increase)	1.06 (0.97–1.18)	1.00 (0.91–1.09)	1.12 (0.96–1.30)
Diastolic BP (per 10 mmHg increase)	1.09 (0.98–1.22)	0.96 (0.86–1.07)	1.17 (0.97–1.42)
Hypertension*	1.41 (1.05–1.89)	1.16 (0.86–1.58)	1.94 (1.10–3.40)
BMI, 3 units increase (kg/m^2^)	1.24 (1.13–1.37)	1.21 (1.10–1.33)	1.25 (1.13–1.38)
<25 kg/m^2^	Ref.	Ref.	Ref.
25–29.9 kg/m^2^	1.40 (1.03–1.90)	1.10 (0.80–1.51)	1.44 (1.00–2.07)
≥30 kg/m^2^	2.08 (1.42–3.05)	1.77 (1.24–2.53)	1.56 (1.23–1.98)
Triglycerides (mmol/L)	1.00 (0.87–1.15)	0.97 (0.83–1.14)	1.00 (0.75–1.33)
Total cholesterol (mmol/L)	1.07 (0.95–1.19)	0.94 (0.84–1.05)	1.13 (0.92–1.39)
Hypercholesterolemia †	1.17 (0.89–1.55)	1.01 (0.77–1.33)	1.36 (0.83–2.23)
HDL cholesterol (mmol/L) ‡	0.97 (0.82–1.14)	0.85 (0.71–1.01)	0.95 (0.76–1.20)
≥1.03 (♂) or ≥1.30 (♀) mmol/L	Ref.	Ref.	Ref.
<1.03 (♂) or <1.30 (♀) mmol/L	0.84 (0.56–1.25)	1.27 (0.91–1.76)	0.73 (0.35–1.51)
Self-reported diabetes	1.43 (0.71–2.91)	1.41 (0.82–2.42)	1.47 (0.69–3.14)
Smoking	1.20 (0.90–1.61)	1.08 (0.79–1.49)	1.30 (0.86–1.96)
Physical activity |	0.98 (0.70–1.36)	1.02 (0.74–1.42)	0.93 (0.34–2.56)
Education ⁞	1.07 (0.75–1.54)	1.10 (0.77–1.57)	1.07 (0.75–1.54)

*Hypertension: systolic BP ≥140 or diastolic BP ≥90 or use of antihypertensive medicine

**†**Hypercholesterolemia: total cholesterol ≥ 6.5 or use of lipid-lowering drugs

**‡**HDL: per 0.5 mmol/L decrease

|Strenuous physical activity 1 hour or more every week

⁞Over/equal to 15 years of education (corresponding to 3 years in university or academy

## Discussion

In the present study, risk estimates for VTE and MI based on one baseline measurement corresponded well with risk estimates based on repeated measurements. Except for BMI, none of the atherosclerotic risk factors were associated with risk of VTE, neither in the time-fixed nor the time-varying model. These results suggest that lack of association between several atherosclerotic risk factors and VTE risk in large prospective cohorts could not be explained by regression dilution bias. For MI, the differences between risk estimates from the time-fixed and the time-varying analysis were greatest for dichotomous variables that changed much during follow-up, such as diabetes, smoking and physical activity. Correction of the time-fixed risk estimates using regression dilution ratios consistently overestimated risk of VTE and MI compared with the time-varying analysis, suggesting that this type of correction should be used with caution.

All the traditional atherosclerotic risk factors were significantly associated with risk of MI in both the time-fixed and time-varying analysis, and the magnitude of the risk estimates corresponded well to those of previous studies [5, 34]. For VTE, only obesity was associated with increased risk also in the time-varying approach. Moreover, the risk estimates were lower in the time-varying than in the time-fixed analyses. This was probably explained by the fact that most subjects experienced small changes in risk factor levels during follow-up, and consequently, those who changed from one risk category to another would most likely contribute to the healthiest part of their new, “unhealthy” category. For example, an individual that changed BMI from 24 to 26 during follow-up would change category from normal weight to overweight but still be under a relatively low risk of MI and VTE.

While BMI has consistently been shown to increase the risk of VTE, the impact of other atherosclerotic risk factors on VTE risk has been controversial [7, 9, 11, 12, 16, 35]. Case-control studies have shown associations between serum lipid levels, diabetes, blood pressure and VTE [11, 13, 15] whereas most cohort studies reported no association [6, 16–18]. While case-control studies may overestimate risks due to reverse causation, recall bias and selected control groups, the potential for regression dilution bias (i.e. underestimation or failure to detect a modest effect that is actually there) has been a major criticism of cohorts with a long follow-up. In the present study, we showed that the degree of regression dilution was very low for most atherosclerotic variables, and that serum lipid levels, smoking, blood pressure and diabetes were not associated with risk of VTE even in the time-varying approach.

Regression dilution was only prominent for yes/no-variables that were strongly associated with MI and had a high degree of intra-individual change during follow-up, such as smoking and physical activity. The percentage of smokers decreased from 33% in Tromsø 4 to 16% in Tromsø 6, and those who stopped smoking during follow-up were misclassified as smokers during the remaining follow-up in the time-fixed model. As these subjects had a reduced risk of MI, the association between smoking and MI was diluted. Additionally, subjects still smoking in Tromsø 6 had smoked for a longer time, and could therefore have been at greater risk of MI. The percentage of physically active subjects increased from 24% in Tromsø 4 to 38% in Tromsø 6, and consequently, the protective effect of physical activity on MI risk was underestimated in the time-fixed analysis.

In regression models with only a single risk factor, the effect of non-differential misclassification is always to reduce the magnitude of the association [19]. However, in multiple regression models, including several risk factors or confounders, non-differential misclassification can actually influence the risk estimates in both directions [36]. In fact, we observed that for some variables the use of a time-varying analysis actually reduced the risk estimates of MI and VTE compared with the time-fixed analysis, whereas Rosner´s method consistently overestimated the risk. The change in risk over time is not only a result of time-dependent covariates, but also influenced by time-dependent effects, and ageing of study participants, change in confounder status, change in environment, or improved treatment could have influenced the effect of exposures over time. For instance, many atherosclerotic risk factors are associated with a higher relative risk of MI in younger adults than in the elderly [37, 38]. In the case of diabetes and risk of MI, the risk was lower in the time varying analysis (HR 2.11) than in the time-fixed analysis (HR 2.94), whereas Rosner´s method showed a substantially higher risk estimate (HR 3.37). The effect of diabetes on the risk of MI varies not only with age and with other confounders, but also with time as the treatment has improved during the last decades. Moreover, newly diagnosed diabetic patients still has a low risk of MI [39, 40], and a higher proportion of newly diagnosed patients than patients with a long-lasting diabetes might additionally explain the lower risk in the time-varying analysis.

Our study supports previous studies finding no association between atherosclerotic risk factors, such as hypertension, serum lipids, diabetes and smoking, and future risk of VTE. The lack of association between serum lipids and VTE suggests that the possible beneficial effects of statins on VTE [41] is mediated through pleiotropic effects rather than the lipid-lowering effect, such as a potential antithrombotic effect [42, 43]. Furthermore, the unfavorable effect of smoking and favorable effect of strenuous physical exercise on the risk of MI are underestimated when single baseline measurements made several years before the event are compared to repeated measurements. Our study support the importance of simple preventive measures, such as smoking cessation, exercise and reducing cholesterol to prevent MI, as previously studied [44, 45].

The main strengths of our study includes the prospective design with participants recruited from a general population, the high attendance rate and the repeated measurements. The repeated measurements allowed us to update the participants’ atherosclerotic risk factors during follow up, and thus reduce misclassification. Furthermore, the municipality of Tromsø is served by a single hospital, minimizing the chance of missing cases and loss to follow-up. Additional use of the National population registry and the National Causes of Death Registry allowed thorough validation of both MI and VTE. The study has some limitations. The study is restricted to a homogenous white population with a certain development of atherosclerotic risk factors over time. Other populations might have different tendencies, and the findings might therefore not apply to all populations. However, it is likely to assume that many of the same trends regarding development in atherosclerotic risk factors are true for most other Western populations.

In conclusion, risk estimates for MI and VTE based on baseline measures and time-fixed analysis corresponded well with risk estimates based on repeated measurements and time-varying analyses. Of the traditional atherosclerotic risk factors, only BMI was associated with VTE in both time-fixed and time-varying analyses, suggesting that underestimation of risks by regression dilution bias is not explaining the lack of association between several atherosclerotic risk factors and VTE risk reported in most prospective cohorts. Our findings suggest that for atherosclerotic risk factors, risk estimates based on a single measurement are generally reliable in cohort studies with long follow-up, and misclassification is a problem only in situations where the association between exposure and outcome is strong and the exposure status varies greatly during follow-up. Correction of the time-fixed risk estimates using the regression dilution ratio consistently overestimated the associations compared to the time-varying analyses.

## Supporting Information

S1 Table Distribution of traditional cardiovascular risk factors in the different studies for participants who participated in all three studies (n = 4391). (DOCX)
