# Supplementary material for: Atherosclerotic Risk Factors and Risk of Myocardial Infarction and Venous Thromboembolism; Time-Fixed versus Time-Varying Analyses. The Tromsø Study
Source: PLoS One. 2016 Sep 16;11(9):e0163242. doi: 10.1371/journal.pone.0163242 (PMC5026338; doi:10.1371/journal.pone.0163242)
Supplement: S1 Table — (DOCX) [file pone.0163242.s001.docx]

**S1 table**. Distribution of traditional cardiovascular risk factors in the different studies for participants who participated in all three studies (n=4391).

| The Tromsø Study | T4 (1994-1995) | T5 (2001-2002) | T6 (2007-2008) |
| --- | --- | --- | --- |
| Age, years | 54.5 ± 10 | 61.5 ± 10 | 67.5 ± 10 |
| Male sex | 39.7 | 39.7 | 39.7 |
| Systolic BP (mmHg) | 138 ± 20 | 140 ± 21 | 145 ± 24 |
| Diastolic BP (mmHg) | 81 ± 12 | 81 ± 12 | 78 ± 11 |
| Hypertension* | 43.8 (1921) | 52.5 (2306) | 67.3 (2953) |
| Antihypertensive treatment | 7.0 (305) | 18.9 (829) | 34.4 (1511) |
| BMI (kg/m^2^) | 25.7 ± 3.7 | 26.7 ± 4.1 | 27.0 ± 4.3 |
| <25 kg/m^2^ | 46.8 (2056) | 34.6 (1520) | 34.2 (1503) |
| 25-29.9 kg/m^2^ | 41.2 (1808) | 44.7 (1964) | 45.2 (1984) |
| ≥30 kg/m^2^ | 11.9 (522) | 20.3 (892) | 20.4 (894) |
| Triglycerides (mmol/L) | 1.55 ± 0.98 | 1.52 ± 0.85 | 1.49 ± 0.81 |
| Total cholesterol (mmol/L) | 6.54 ± 1.26 | 6.30 ± 1.16 | 5.73 ± 1.13 |
| Hypercholesterolemia† | 50.7 (2225) | 50.5 (2217) | 46.9 (2058) |
| Lipid lowering drugs | 1.3 (56) | 11.3 (497) | 22.6 (992) |
| HDL cholesterol (mmol/L) | 1.57 ± 0.42 | 1.49 ± 0.40 | 1.57 ± 0.46 |
| ≥1.03 (♂) or ≥1.30 (♀) mmol/L | 84.2 (3698) | 78.4 (3442) | 82.4 (3616) |
| <1.03 (♂) or <1.30 (♀) mmol/L | 15.5 (681) | 21.2 (931) | 16.4 (722) |
| Self-reported diabetes | 1.3 (57) | 3.1 (137) | 6.9 (302) |
| Smoking | 29.9 (1314) | 24.8 (1089) | 16.4 (719) |
| Physical activity\| | 23.6 (1135) | 28.4 (1245) | 31.5 (1383) |
| Education⁞ | 21.7 (953) | 21.7 (954) | 23.6 (1937) |

Values are % (n) or mean±SD
*Hypertension: systolic BP > 140 or diastolic BP > 90 or use of antihypertensive medicine
**†**Hypercholesterolemia: total cholesterol ≥ 6.5 or use of lipid-lowering drugs
|Strenuous physical activity 1 hour or more every week
⁞Over/equal to 15 years of education (corresponding to 3 years in university or academy)
